# Effect of Topical Prostaglandin F2α Analogs on Selected Oxidative Stress Parameters in the Tear Film

**DOI:** 10.3390/medicina55070366

**Published:** 2019-07-11

**Authors:** Lech Sedlak, Maria Zych, Weronika Wojnar, Dorota Wyględowska-Promieńska

**Affiliations:** 1 Department of Ophthalmology, School of Medicine in Katowice, Medical University of Silesia in Katowice, 40-514 Katowice, Poland; 2 Department of Ophthalmology, Kornel Gibiński University Clinical Center, Medical University of Silesia in Katowice, 40-514 Katowice, Poland; 3 Department of Pharmacognosy and Phytochemistry, School of Pharmacy with the Division of Laboratory Medicine, Medical University of Silesia in Katowice, 41-200 Sosnowiec, Poland

**Keywords:** glaucoma, ocular surface, benzalkonium chloride, latanoprost, tafluprost, bimatoprost, tears

## Abstract

*Background and Objectives*: Topically administered antiglaucoma medications, especially those containing benzalkonium chloride (BAC), may cause local adverse effects and compromise ocular surface. The aim of the study was to assess the effect of topical prostaglandin F2α analogs (PGAs): preservative-free latanoprost, BAC-preserved latanoprost, preservative-free tafluprost, and BAC-preserved bimatoprost, on selected oxidative stress parameters in the tear film. *Materials and Methods*: The patients were divided into five groups: group C (*n* = 25) control group—subjects who did not use topical antiglaucoma medications, group L (*n* = 22)—patients using topical preservative-free latanoprost, group L+BAC (*n* = 25)—patients using topical BAC-preserved latanoprost, group T (*n* = 19)—patients using topical preservative-free tafluprost, and group B+BAC (*n* = 17)—patients using topical BAC-preserved bimatoprost. The oxidative stress markers in the tear film samples were evaluated: total protein (TP) concentration, advanced oxidation protein products (AOPP) content, total sulfhydryl (-SH) groups content, the activity of superoxide dismutase (SOD), catalase (CAT), and glutathione peroxidase (GPx), as well as Total Oxidant Status (TOS), Total Antioxidant Response (TAR), and Oxidative Stress Index (OSI). *Results*: The TP concentrations in the groups L, L+BAC, and B+BAC were statistically significantly higher in comparison with group C. The SOD and CAT activities in the groups L+BAC and B+BAC were statistically significantly higher when compared to group C. As compared to group C, AOPP and TOS were statistically significantly higher in all the study groups. OSI was found to be statistically significantly higher in the groups L+BAC, T, and B+BAC in comparison with group C. *Conclusion*: Use of topical PGAs by the patients with ocular hypertension or primary open-angle glaucoma is associated with increased oxidative stress in the tear film which is additionally exacerbated by the presence of BAC in the formulation.

## 1. Introduction

Glaucoma is a disorder of the optic nerve characterized by accelerated apoptosis of the ganglion cells and nerve fibers leading to the gradual loss of visual field. The main risk factor of glaucomatous neuropathy is elevated intraocular pressure (IOP). Most of the patients are treated by topical IOP-lowering medications. If effective, these drugs are usually recommended indefinitely, and therefore the majority of patients remain under lifelong medical therapy. According to the European Glaucoma Society (EGS), the goal of the treatment is to maintain the patient’s visual function and related quality of life, at a sustainable cost. This implicates minimization of the side effects of the treatment [1]. However, prostaglandin F2α analogs (PGAs), which are the first-line eye drops used in primary open-angle glaucoma (POAG), commonly cause local side effects such as burning, stinging, foreign body sensation, tearing, or conjunctival hyperemia, especially in patients undergoing long-term treatment [2,3]. These adverse effects not only reduce the quality of life but are also responsible for treatment cessation by the patients.

The preservatives have been required in the multidose eye drop formulations for the prevention of microbial proliferation. Benzalkonium chloride (BAC), which has been used in ophthalmology since the 1940s, is the most common preservative. This quaternary ammonium shows highly effective antibacterial and antifungal properties achieved by lysing cell membranes of microorganisms [4]. However, this compound was found by numerous studies to cause destabilization of the tear film and corneal and conjunctival epithelial cell damage which, in turn, escalates the inflammation of the ocular surface [5].

So far, a direct impact of topical PGAs, both BAC-preserved and nonpreserved, on oxidative stress in the tear film was not investigated.

Therefore, the aim of this study was to assess the effect of topical PGAs: preservative-free latanoprost, BAC-preserved latanoprost, preservative-free tafluprost, and BAC-preserved bimatoprost, on total protein (TP) concentration, advanced oxidation protein products (AOPP) content, total sulfhydryl (-SH) groups content, the activity of superoxide dismutase (SOD), catalase (CAT), and glutathione peroxidase (GPx), as well as Total Oxidant Status (TOS), Total Antioxidant Response (TAR), and Oxidative Stress Index (OSI) in the tear film.

## 2. Materials and Methods

This cross-sectional study was approved by the Bioethical Committee of Medical University of Silesia (approval number: KNW/0022/KB1/87/17). The performance of this study was conducted in accordance with the ethical standards laid down in the Declaration of Helsinki (Seventh revision, October 2013, Fortaleza, Brazil).

The study was conducted among the patients of the outpatient clinic as well as the patients of the Department of Ophthalmology, Kornel Gibiński University Clinical Center, Medical University of Silesia in Katowice, Poland. For study groups, the patients older than 18 and younger than 70 years of age using single topical prostaglandin F2α analog were recruited. A general medical and ophthalmic history (including self-answered Ocular Surface Disease Index—OSDI) was gained and a basic slit-lamp biomicroscope eye examination was carried out.

The exclusion criteria were as follows:

- The use of any other topical medication
- The use of fixed-combination antiglaucoma medications
- The history of diabetes and autoimmune, allergic, and thyroid disorders
- The history of dry eye disease (OSDI score >12)
- The history of refractive and intraocular surgery as well as recent oculoplastic procedures
- The signs of anterior segment inflammation (keratitis, conjunctivitis, uveitis) and meibomian gland dysfunction
- The features of ocular surface disease in the slit-lamp eye examination (including the presence of LIPCOF—lid-parallel conjunctival folds, corneal superficial punctate fluorescein staining, and FBUT—fluorescein break-up time >10 s).

In total, 108 patients were recruited and divided into five groups:

- group C (*n* = 25), control group—subjects who did not use topical antiglaucoma medications
- group L (*n* = 22), patients with glaucoma or ocular hypertension using topical preservative-free latanoprost (Monoprost, Thea Laboratoires)
- group L+BAC (*n* = 25), patients with glaucoma or ocular hypertension using topical BAC-preserved latanoprost (Xalatan, Pfizer)
- group T (*n* = 19), patients with glaucoma or ocular hypertension using topical preservative-free tafluprost (Taflotan, Santen)
- group B+BAC (*n* = 17), patients with glaucoma or ocular hypertension using topical BAC-preserved bimatoprost (Lumigan, Allergan)

Subjects in the Groups C, L, L+BAC, T, and B+BAC had been using the eye drops once daily for 6–12 months.

Saline in the amount of 30 µL was pipetted to the eye followed by moving closed eye by the patient for 5 s. Afterward, a 40 µL glass capillary micropipette was placed temporally at the lower tear meniscus and the tears were collected via capillary action. The samples of the tear film were subsequently transferred into 1 mL Eppendorf tubes followed by immediate freezing at −80 °C [6].

The oxidative stress markers in the tear film samples were evaluated: TP concentration, AOPP content, total -SH groups content, the activity of SOD, CAT, and GPx, as well as TOS, TAR, and OSI.

The activity of antioxidant enzymes, including SOD, CAT, and GPx, was assessed using commercially available kits (Cayman Chemical, Ann Arbor, MI, USA). The results were standardized on protein content, estimated by colorimetric kit (BioSystems S.A. Costa Brava, Barcelona, Spain).

Ellman’s method was used in order to evaluate total -SH groups in the tears [7]. Briefly, to all samples, phosphate buffer (pH = 8) and 5,5′-dithiobis-(2-nitrobenzoic acid) were added. After color development, all probes were measured at 412 nm and then calculated with extinction coefficient = 13,600 M/cm.

The content of AOPP in the tears was evaluated using a method described by Witko-Sarsat et al. [8]. In this method, the aliquot of the tear sample was mixed with potassium iodide (1.16 M) and glacial acetic acid. All samples and standard curve prepared with chloramine T were measured at 340 nm. Similar to the antioxidant enzymes, the results were standardized on protein content.

TAR was assayed using Erel’s method [9]: the samples were mixed with reagent 1, composed of o-dianisidine and ferrous ions dissolved in Clark and Lubs solution, then read at 444 nm. Afterward, reagent 2 (hydrogen peroxide in Clark and Lubs solution) was added and after 4 min of incubation, all samples were read at 444 nm. The difference between measurement 2 and 1 represented TAR value. The results are presented as Trolox equivalent/mL of tears. Total oxidative status was estimated according to Erel’s protocol [10]: to the probe, a mixture of xylenol orange with NaCl and glycerol in H_2_SO_4_ solution (reagent 1) was added and first measurement (at 560 and 800 nm as reference) was taken. Subsequently, the reagent 2 (consisting of ferrous ions and o-dianisidine in H_2_SO_4_ solution) was added and after 4 min of incubation, the second measurement at 560 and 800 nm was added. The results from measurement 1 were extracted from those from measurement 2. The obtained difference represented TOS values for the probes. Results are presented as H_2_O_2_ equivalent per 1 mL of the tears. Values for TAR and TOS were used to calculate the Oxidative Stress Index (OSI) according to the following equation: OSI = TOS/TAR [11].

The obtained results were subjected to statistical analysis with one-way ANOVA followed by Duncan’s post hoc test in Statistica 10 Software (StatSoft, Tulsa, OK, USA). All results are presented as arithmetic mean ± SEM. Results were considered statistically significant if the *p*-values were <0.05.

## 3. Results

### 3.1. Effect of Topical Prostaglandin F2α Analogs on the TP Concentration in the Tear Film

**Figure 1:**
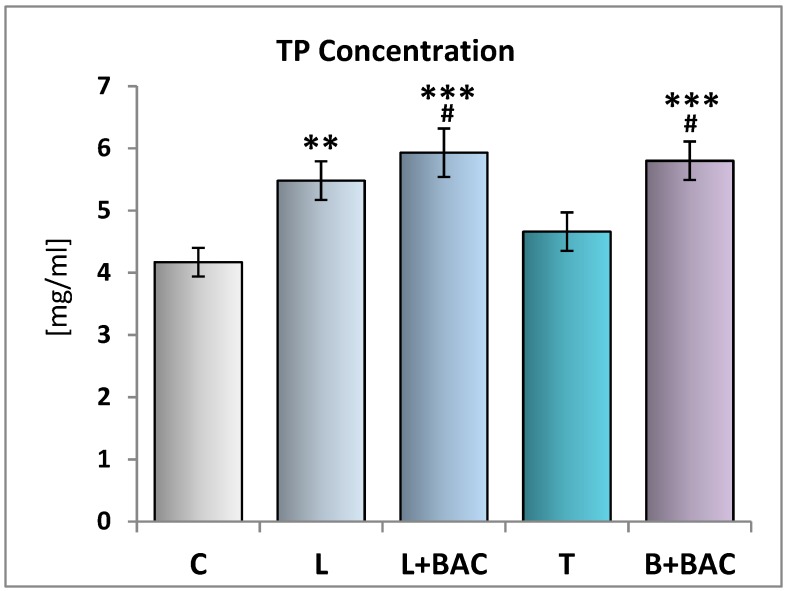
Effect of topical prostaglandin F2α analogs on the total protein (TP) concentration in the tear film. C—control group (*n* = 25); L—patients with glaucoma or ocular hypertension using topical preservative-free latanoprost (Monoprost, Thea Laboratoires) (*n* = 22); L+BAC (benzalkonium chloride)—patients with glaucoma or ocular hypertension using topical BAC-preserved latanoprost (Xalatan, Pfizer) (*n* = 25); T—patients with glaucoma or ocular hypertension using topical preservative-free tafluprost (Taflotan, Santen) (*n* = 19); B+BAC—patients with glaucoma or ocular hypertension using topical BAC-preserved bimatoprost (Lumigan, Allergan) (*n* = 17). Results are presented as means ± SEM. ** *p* < 0.01, *** *p* < 0.001—statistically significantly different from the C group. # *p* < 0.05—statistically significantly different from the T group.

In the control group (group C), the TP concentration in the tear film was 4.17 ± 0.23 mg/mL. In comparison with group C, the TP concentration in the group of patients using topical preservative-free latanoprost (group L) was statistically significantly higher (*p* < 0.01). The TP concentration in the group of patients using topical BAC-preserved latanoprost (group L+BAC) was statistically significantly higher (*p* < 0.001) as compared to group C, as well as higher than in group L (not statistically significantly). In the group of patients using topical preservative-free tafluprost (group T), the TP concentration was higher when compared with group C, whereas it was lower in comparison with group L (both not statistically significantly). The TP concentration in the group of patients using topical BAC-preserved bimatoprost (group B+BAC) was statistically significantly higher as compared to group C (*p* > 0.001). However, no significant differences were noted in comparison with the results obtained in the groups L and L+BAC (Figure 1).

### 3.2. Effect of Topical Prostaglandin F2α Analogs on the Enzymatic Oxidative Stress Parameters in the Tear Film

**Figure 2:**
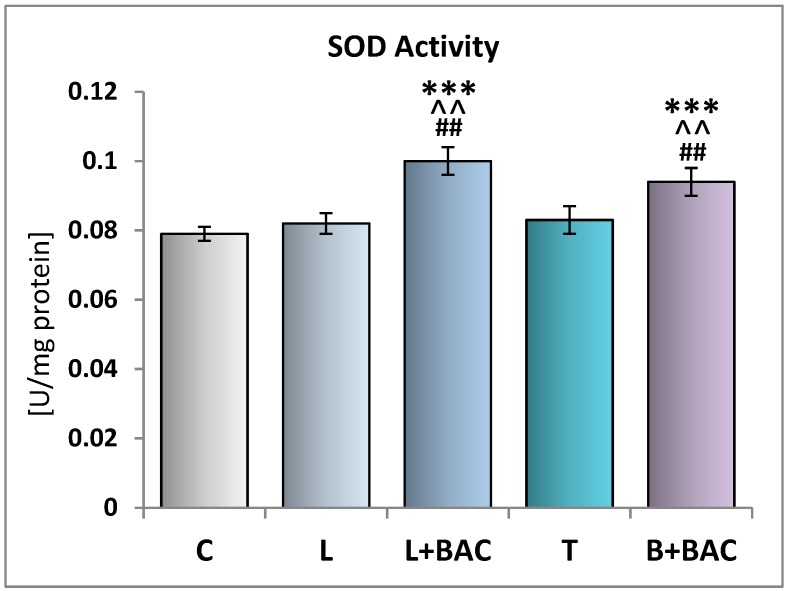
Effect of topical prostaglandin F2α analogs on the superoxide dismutase (SOD) activity in the tear film. C—control group (*n* = 25); L—patients with glaucoma or ocular hypertension using topical preservative-free latanoprost (Monoprost, Thea Laboratoires) (*n* = 22); L+BAC (benzalkonium chloride)—patients with glaucoma or ocular hypertension using topical BAC-preserved latanoprost (Xalatan, Pfizer) (*n* = 25); T—patients with glaucoma or ocular hypertension using topical preservative-free tafluprost (Taflotan, Santen) (*n* = 19). B+BAC—patients with glaucoma or ocular hypertension using topical BAC-preserved bimatoprost (Lumigan, Allergan) (*n* = 17). Results are presented as means ± SEM. *** *p* < 0.001—statistically significantly different from the C group. ^^ *p* < 0.01—statistically significantly different from the L group. ## *p* < 0.01—statistically significantly different from the T group.

The activity of antioxidative enzymes SOD, CAT, GPx in the control group (group C) was 0.079 ± 0.002 U/mg of protein, 0.6 ± 0.03 nmol/min/mg of protein, and 3.72 ± 0.24 nmol/min/mg of protein, respectively. There were no significant differences with regard to the SOD activities between groups C, L, and T. However, in comparison with group C, in groups of patients using topical medications containing BAC (groups L+BAC and B+BAC), the SOD activities were statistically significantly higher (*p* < 0.001). Moreover, in these groups, the activities of SOD were statistically significantly higher (*p* < 0.01) when confronted with the tear film of patients using preservative-free medications (groups L and T). No difference in this parameter was recorded between groups with BAC-preserved eye drops (groups L+BAC and B+BAC) (Figure 2).

**Figure 3:**
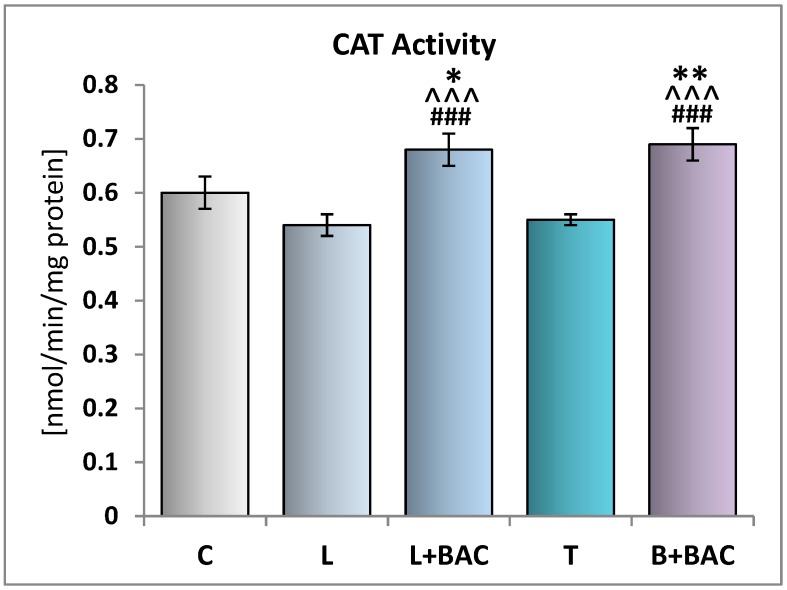
Effect of topical prostaglandin F2α analogs on the catalase (CAT) activity in the tear film. C—control group (*n* = 25); L—patients with glaucoma or ocular hypertension using topical preservative-free latanoprost (Monoprost, Thea Laboratoires) latanoprost (*n* = 22); L+BAC (benzalkonium chloride)—patients with glaucoma or ocular hypertension using topical BAC-preserved latanoprost (Xalatan, Pfizer) (*n* = 25); T—patients with glaucoma or ocular hypertension using topical preservative-free tafluprost (Taflotan, Santen) (*n* = 19); B+BAC—patients with glaucoma or ocular hypertension using topical BAC-preserved bimatoprost (Lumigan, Allergan) (*n* = 17). Results are presented as means ± SEM. * *p* < 0.05, ** *p* < 0.01—statistically significantly different from the C group. ^^^ *p* < 0.001—statistically significantly different from the L group. ### *p* < 0.001—statistically significantly different from the T group.

Regarding CAT activities, there were no significant differences between groups C, L, and T. Similarly to SOD, CAT activities in the tear film of patients receiving BAC-containing medications (groups L+BAC and B+BAC) were statistically significantly higher, as compared to the control group (*p* < 0.05 and *p* < 0.01, respectively). This parameter was also statistically significantly higher (*p* < 0.001) in groups L+BAC and B+BAC when compared to the groups of subjects using preservative-free topical prostaglandin F2α analogs (groups L and T). The CAT activities were similar between the BAC-preserved latanoprost (group L+BAC) and bimatoprost (group B+BAC) (Figure 3).

**Figure 4:**
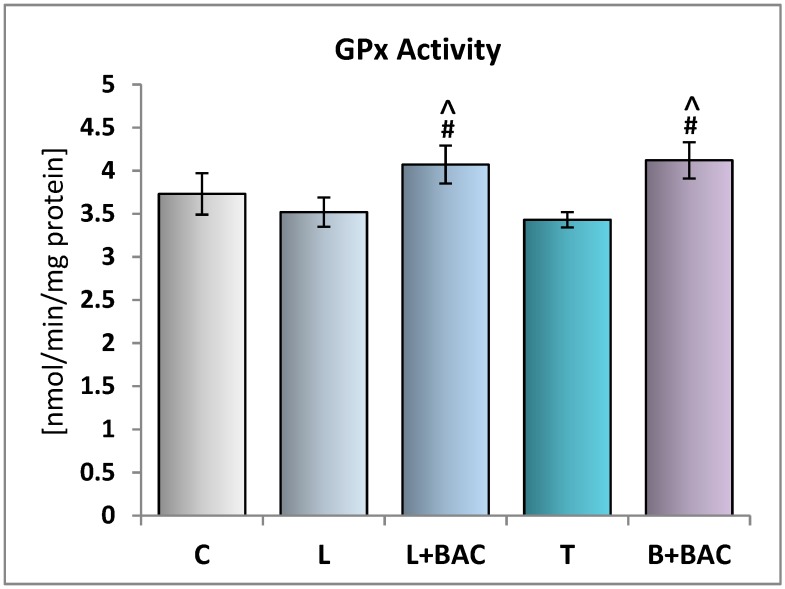
Effect of topical prostaglandin F2α analogs on the glutathione peroxidase (GPx) activity in the tear film. C—control group (*n* = 25); L—patients with glaucoma or ocular hypertension using topical preservative-free latanoprost (Monoprost, Thea Laboratoires) (*n* = 22); L+BAC (benzalkonium chloride)—patients with glaucoma or ocular hypertension using topical BAC-preserved latanoprost (Xalatan, Pfizer) (*n* = 25); T—patients with glaucoma or ocular hypertension using topical preservative-free tafluprost (Taflotan, Santen) (*n* = 19); B+BAC—patients with glaucoma or ocular hypertension using topical BAC-preserved bimatoprost (Lumigan, Allergan) (*n* = 17). Results are presented as means ± SEM. ^ *p* < 0.05—statistically significantly different from the L group. # *p* < 0.05—statistically significantly different from the T group.

The GPx activity was not significantly different between the control group and the groups of the tear film of patients with preservative-free medications (group L and T). This parameter was not statistically significantly higher in the groups L+BAC and B+BAC in comparison with group C. However, with regard to GPx activities, we noted statistically significant differences between the groups of subjects receiving BAC-containing medications (groups L+BAC and B+BAC) when confronted with groups of preservative-free antiglaucoma eye drops (groups L and T, *p* < 0.05) (Figure 4).

### 3.3. Effect of Topical Prostaglandin F2α Analogs on the Nonenzymatic Oxidative Stress Parameters Content in the Tear Film

The AOPP content in the control group (group C) was 3.64 ± 0.21 nmol/min/mg of protein. In comparison with group C, the AOPP content was found to be statistically significantly higher in the groups L (*p* < 0.05), L+BAC (*p* < 0.01), T (*p* < 0.01), and B+BAC (*p* < 0.01). There were no significant differences between the study groups in terms of the AOPP content (Table 1).

The total -SH groups content in the control group was 0.731 ± 0.018 µmol/mL, while in all study groups (groups L, L+BAC, T, and B+BAC), it was statistically significantly lower (*p* < 0.05, *p* < 0.01, *p* < 0.05, and *p* < 0.05, respectively). No significant differences with regard to this parameter between the groups L, L+BAC, T, and B+BAC were recorded (Table 1).

### 3.4. Effect of Topical Prostaglandin F2α Analogs on the Total Oxidant Status (TOS), Total Antioxidant Reactivity (TAR), and Oxidative Stress Index (OSI) in the Tear Film

In the control group, the TOS was 0.069 ± 0.003 nmol H_2_O_2_/mL. When compared to this group, the TOS was found to be statistically significantly higher in the groups L (*p* < 0.01), L+BAC (*p* < 0.001), T (*p* < 0.001), and B+BAC (*p* < 0.001) (Table 2).

The TAR in the control group was 0.066 ± 0.003 (µmol trolox/mL), while in all other groups, this parameter was slightly higher (not statistically significantly) (Table 2).

The OSI was recorded to be 1.05 ± 0.02 in group C. As compared to the result obtained in this group, the OSI in group L was not significantly higher, whereas in the groups L+BAC, T, and B+BAC, this index was statistically significantly higher (*p* < 0.001, *p* < 0.01, *p* < 0.05, respectively) (Table 2).

## 4. Discussion

The prostaglandin F2α analogs (PGAs) are the first-choice treatment for ocular hypertension and POAG as approved by the EGS due to the effective reduction of intraocular pressure and lack of severe systemic side effects [1]. However, like other topical IOP-lowering medications, these eye drops may produce local side effects such as foreign body sensation, burning, stinging, or conjunctival hyperemia. They are especially frequent in patients under long-term treatment, reducing the patients' quality of life and commonly being a cause of treatment discontinuation. The symptoms and signs of these side effects are consistent with the clinical features of dry eye syndrome (DES). Wong et al. have demonstrated significantly poorer noninvasive tear film breakup time, tear film osmolarity, tear meniscus height, and anesthetized Schirmer value in eyes treated with topical antiglaucomatous medications when compared with fellow eyes [12]. Specific side effects of PGAs are increased eyelash growth, iris and periocular pigmentation, and hypertrichosis [13].

Preservatives are substances added to topical medications in order to prevent microbial proliferation after opening the bottle. The most commonly used preservative in eye drops is BAC. This chemical is a quaternary ammonium acting by dissociation of cellular membrane layers which, in turn, induces leakage of bacterial cellular contents [5].

In a large cross-sectional epidemiologic study, the patients using preserved beta-blocking eye drops have more frequently reported local side effects than the users of preservative-free medications, such as pain or discomfort during instillation (48 vs. 19%, respectively), foreign body sensation (42 vs. 15%), stinging or burning (48 vs. 20%), and dry eye sensation (35 vs. 16%) [14]. It has also been observed that after switching from BAC-preserved topical antiglaucoma medications to preservative-free eye drops, the ocular surface and symptoms improve significantly. Uusitalo et al. have demonstrated a decrease in the occurrence of irritation/burning/stinging, foreign body sensation, tearing, itching, dry eye sensation, blepharitis, corneal/conjunctival fluorescein staining 12 weeks after changing therapy from preserved latanoprost to preservative-free tafluprost [2]. Similar results were received by the RELIEF study in which the tolerability and efficacy following a switch from BAC-preserved latanoprost to preservative-free latanoprost were assessed [3].

The detrimental actions of BAC exerted on the ocular surface have been widely described and proven by numerous in vitro, in vivo, as well as clinical studies. Its cytotoxic threshold has been established at the concentration of 0.005%, which is lower than in most of the topical medications [5]. For example, the investigators found that an exposure of the mucous layer of the tear film to 0.01% BAC for 5 or 15 min caused its fixation while prolonged exposure (60 min) destroyed that layer [15]. In an animal model, the administration of latanoprost with 0.02% BAC resulted in lower densities of goblet cells compared with the use of either SofZia-preserved travoprost or preservative-free artificial tears. However, the authors of this study did not assess the influence of latanoprost alone [16]. Furthermore, being a tensioactive compound, this preservative disturbs the integrity of the lipid layer of the tear film causing excessive tear evaporation [5]. In addition, numerous studies show the direct proinflammatory impact of BAC mediated by the release inflammatory cytokines as well as overexpression of receptors to cytokines of chemokines such as CX3CL1, CCR4, and CCR5 [17,18]. Another harmful mechanism of BAC is the promotion of hyperosmolarity, which is a repeatable and objective feature of DES [19]. There is a significant positive correlation between the number of preserved antiglaucomatous medications used by the patient and the tear osmolarity [20]. Tear film hyperosmolarity induces the secretion of inflammatory mediators leading to further loss of goblet cells and to epithelial ocular surface damage. This exacerbates the destabilization of the tear film and propagates the vicious cycle of dry eye. A recent in vitro study on an immortalized human meibomian gland epithelial cell line showed the cytotoxic effect of BAC, but also indicated a synergistic influence of the combination of this preservative and prostaglandin analogs resulting in higher toxicity [21].

Despite widely described unfavorable actions of BAC and PGAs, a direct influence of preserved and unpreserved PGAs on oxidative stress in the tear film was not investigated so far.

Oxidative stress is a phenomenon of disturbance between the production and accumulation of reactive oxidative species (ROS) and the ability of the biological system to neutralize these reactive products. Biological antioxidant defenses include enzymatic and nonenzymatic antioxidants that are usually effective in preventing deleterious effects of ROS. However, in pathological conditions, they can be overwhelmed by high ROS concentrations, which damage cell structures such as carbohydrates, nucleic acids, lipids, and proteins and disturb their functions. Various factors may potentially increase the amount of ROS on the ocular surface and tear film such as ultraviolet (UV) light, chemical compounds and pollutants, microbial antigens, hormone and age-related factors. Oxidative stress is also considered to be one of the components of the pathophysiological vicious cycle of DES [22]. Deng et al. have reported that the hyperosmolarity leads to a significant increase in ROS production along with oxidative stress markers such as 4–hydroxynonenal, malondialdehyde, and 8-Oxo-2′-deoxyguanosine, as well as reduction of SOD1 and GPx levels in cultured primary human corneal epithelial cells (HCECs) [23].

The tear film and ocular surface contain antioxidant defenses in the form of antioxidative enzymes present in the aqueous layer–SOD, CAT, GPx, as well as nonenzymatic antioxidants such as glutathione, ascorbic acid, lactoferrin, uric acid, and cysteine [24,25,26]. To the best of our knowledge, the presence of AOPP, as well as the content of total -SH groups in the tear film, were first determined in our study.

The present study was conducted among healthy individuals who did not use any eye drops and among the patients with POAG or ocular hypertension using BAC-preserved or preservative-free topical PGAs for 6 to 12 months. The patients recruited to our study declared that they were using PGAs 6 to 12 months as the only eye drops, however, we could not verify if every participant of the study made true and correct statements. Another limitation of this study is the possibility of noncompliance with the recommendation of daily use of medication or improper technique for applying eye drops by some of the patients, leading to lack of activity of the drug. We believe that 6 months are sufficient to observe changes in the levels of oxidative stress parameters. Also, the patients using eye drops longer than 12 months were excluded since the duration of local IOP-lowering treatment is a risk factor for the development of DES [27]. We also took care to minimize the factors that may influence the oxidative stress in the tear film. For this reason, we excluded the patients with symptoms and signs of DES, the signs of anterior segment inflammation and meibomian gland dysfunction, the patients after recent refractive, intraocular, and oculoplastic procedures, as well as the patients with diabetes, autoimmune, and allergic disorders in their medical history. Furthermore, the persons older than 70 years of age were excluded from the study since aging is associated with increased oxidative stress and a significantly higher prevalence of ocular surface diseases [27,28].

In our study, we noted significantly higher TOS and OSI in all the groups of patients treated with PGAs, both BAC-preserved and preservative-free formulations, indicating increased oxidative stress in the tear film. The oxidative stress in these groups may be a consequence of the reduction in the content of the total -SH groups. Yet, only in the cases of the tear film of patients using BAC-preserved PGAs, the oxidative stress was demonstrated by increased activity of SOD, CAT, and GPx. The elevated activity of antioxidative enzymes may be a result of intensified defense of the ocular surface against oxidative stress. Other investigators have found lower levels of CAT and SOD in the tears of patients treated with preserved sodium hyaluronate 0.1% and preserved fluorometholone 0.1% eye drops following cataract surgery when compared to the patients using preservative-free preparations of these medications [29]. The differences in the oxidative stress parameters in our study between the patients treated with BAC-preserved PGAs and those treated with nonpreserved PGAs indicate a different mechanism of oxidative stress promoted by the active ingredient of the medications and by the preservative. However, in all the study groups, we detected an increased AOPP as a consequence of oxidative stress.

In the present study, the levels of oxidative stress parameters changed in the tear film of patients both using BAC-preserved and preservative-free eye drops. This suggests that the oxidative stress on the ocular surface is caused not only by the preservative but also by active ingredients of the medications. After all, prostaglandin receptors are known to contribute to inflammatory and allergic responses. For example, the activation of PGF2α receptors facilitates pulmonary fibrosis independently of TGF-beta after microbial invasion [30]. Other reports showed that the biosynthesis of PGF2α is elevated in patients with rheumatoid arthritis, psoriatic arthritis, reactive arthritis, and osteoarthritis [31].

Also, an elevation in PGF2α metabolites in body fluids was found to be variably associated with the presence of cardiovascular risk factors such as diabetes, obesity, smoking, as well thickening of intima–media ratio in the carotid artery [32,33]. Furthermore, it has been suggested that the acute inflammation evoked biosynthesis of PGF2α may be related to free radical-induced generation of F ring isoprostanes, which are the markers of lipid peroxidation [34,35]. The PGF2α also plays a role in allergic responses. Its presence in human sputum was shown to be connected with sputum eosinophil levels and causes airway constriction [36].

Interestingly, in our study, PGAs have increased the TP concentration in the tear film. We are not able to entirely explain the cause of this observation. However, PGAs were shown to promote matrix metalloproteinases (MMPs) expression. Specifically, overexpression of MMP-1 and MMP-9 was demonstrated in the corneal stroma by immunohistochemical staining in the rabbits treated with PGAs. The same investigators detected an elevated mRNA of MMP-1, MMP-3, and MMP-9 in the conjunctival epithelial cells along with increased levels of MMP-1, MMP-3, and MMP-9 in the tears of PGA-treated humans [37]. MMPs are the important proteins engaged in extracellular matrix homeostasis as well as other physiological or pathological processes. They are also expressed due to inflammatory stimulus or disturbance of the ocular surface. For instance, an elevated MMP-9 is detected in the tears of subjects with ocular surface diseases such as keratoconus, ocular allergy, and DES [38,39,40]. In a recent study, Reddy et al. have reported higher expression of MMP-2 in tears of eyes receiving bimatoprost and higher MMP-9 in eyes receiving latanoprost, as well as elevated cytokines regulating allergic pathways in eyes treated with bimatoprost [41]. Therefore, the local side effects of PGAs may be partly attributed to the upregulation of the MMPs and cytokines induced by these drugs.

Regardless of the preservatives, the impact of other excipients contained in the topical medications such as surfactants/cosolubilizers on the oxidative stress and ocular surface cannot be excluded, however, their significance is probably lower [42].

Our study confirms the need to assess the therapeutic potential of antioxidants in the form of eye drops to minimize the oxidative stress on the ocular surface. Such investigations are already being conducted. For instance, Cavet et al. demonstrated a decrease of ROS levels after treatment of HCECs with green tea epigallocatechin gallate [43]. In another study of in vitro culture model of HCECs, blueberry component pterostilbene was found to suppress ROS overproduction in a dose-dependent manner, reduce the levels of malondialdehyde, 4-hydroxynonenal, aconitase-2, and 8-hydroxydeoxyguanosine, as well as restore the activity of SOD and reduce the expression of proinflammatory mediators [44].

## 5. Conclusions

The higher TOS, OSI, and AOPP content, as well as lower total -SH groups content, indicate increased oxidative stress in the tear film of patients using topical preservative-free PGAs: latanoprost and tafluprost. Similar changes in the tear film were observed in the patients treated with BAC-preserved latanoprost and BAC-preserved bimatoprost. Additionally, increased activity of SOD, CAT, and GPx in the tear film of these patients suggest an intensification of oxidative stress by BAC. Furthermore, using these topical drugs leads to an elevation of the TP concentration in the tear film, regardless of the presence of BAC.

The obtained results may indicate an increased engagement of the ocular surface defense system of the patients using topical PGAs. Undoubtedly, these medications are not neutral to the ocular surface. It can be speculated that the changed oxidative stress parameters are the first sign of the disturbance and the further prolonged use of the topical PGAs leads to the exacerbation of oxidative stress manifestation of the local side effects of these drugs such as inflammation and/or DES. However, so far, very few studies related to the antioxidant system of the tear film have been performed. Therefore, at this stage, it is difficult to make long-range conclusions on the relation of the oxidative stress and the adverse effects of topical antiglaucoma medications.

## Figures and Tables

**Table 1:** Effect of topical prostaglandin F2α analogs on the advanced oxidation protein products (AOPP) and total sulfhydryl (-SH) groups content in the tear film.

Parameter/Group	C	L	L+BAC	T	B+BAC
**AOPP** (nmol/mg protein)	3.64 ± 0.21	4.17 ± 0.13 *	4.39 ± 0.2 **	4.46 ± 0.15 **	4.33 ± 0.18 **
**Total -SH groups** (µmol/mL)	0.731 ± 0.018	0.675 ± 0.014 *	0.670 ± 0.014 **	0.680 ± 0.013 *	0.676 ± 0.013 *

C—control group (*n* = 25); L—patients with glaucoma or ocular hypertension using topical preservative-free latanoprost (Monoprost, Thea Laboratoires) (*n* = 22); L+BAC (benzalkonium chloride)—patients with glaucoma or ocular hypertension using topical BAC-preserved latanoprost (Xalatan, Pfizer) (*n* = 25); T—patients with glaucoma or ocular hypertension using topical preservative-free tafluprost (Taflotan, Santen) (*n* = 19); B+BAC—patients with glaucoma or ocular hypertension using topical BAC-preserved bimatoprost (Lumigan, Allergan) (*n* = 17). Results are presented as means ± SEM. * *p* < 0.05, ** *p* < 0.01—statistically significantly different from the C group.

**Table 2:** Effect of topical prostaglandin F2α analogs on the Total Oxidant Status (TOS), Total Antioxidant Reactivity (TAR), and Oxidative Stress Index (OSI) in the tear film.

Parameter/group	C	L	L+BAC	T	B+BAC
**TOS** (nmol H_2_O_2_/mL)	0.069 ± 0.003	0.080 ± 0.002 **	0.085 ± 0.003 ***	0.087 ± 0.003 ***	0.085 ± 0.004 ***
**TAR** (µmol trolox/mL)	0.066 ± 0.003	0.072 ± 0.004	0.070 ± 0.002	0.073 ± 0.003	0.072 ± 0.002
**OSI**	1.05 ± 0.02	1.15 ± 0.04	1.29 ± 0.05 ***^	1.22 ± 0.05 **	1.19 ± 0.05 *

C—control group (*n* = 25); L—patients with glaucoma or ocular hypertension using topical preservative-free latanoprost (Monoprost, Thea Laboratoires) (*n* = 22); L+BAC (benzalkonium chloride)—patients with glaucoma or ocular hypertension using topical BAC-preserved latanoprost (Xalatan, Pfizer) (*n* = 25); T—patients with glaucoma or ocular hypertension using topical preservative-free tafluprost (Taflotan, Santen) (*n* = 19); B+BAC—patients with glaucoma or ocular hypertension using topical BAC-preserved bimatoprost (Lumigan, Allergan) (*n* = 17). Results are presented as means ± SEM. * *p* < 0.05, ** *p* < 0.01, *** *p* < 0.001—statistically significantly different from the C group. ^ *p* < 0.05—statistically significantly different from the L group.

## References

[1] (2017). European Glaucoma Society Terminology and Guidelines for Glaucoma, 4th Edition-Chapter 3: Treatment principles and options. Br. J. Ophthalmol..

[2] Uusitalo H., Egorov E., Kaarniranta K., Astakhov Y., Ropo A. (2016). Benefits of switching from latanoprost to preservative-free tafluprost eye drops: A meta-analysis of two Phase IIIb clinical trials. Clin. Ophthalmol..

[3] Misiuk-Hojlo M., Pomorska M., Mulak M., Rekas M., Wierzbowska J., Prost M., Wasyluk J., Lubinski W., Podboraczynska-Jodko K., Romaniuk W. (2018). The RELIEF study: Tolerability and efficacy of preservative-free latanoprost in the treatment of glaucoma or ocular hypertension. Eur. J. Ophthalmol..

[4] Steven D.W., Alaghband P., Lim K.S. (2018). Preservatives in glaucoma medication. Br. J. Ophthalmol..

[5] Baudouin C., Labbé A., Liang H., Pauly A., Brignole-Baudouin F. (2010). Preservatives in eyedrops: the good, the bad and the ugly. Prog. Retin. Eye Res..

[6] Ablamowicz A.F., Nichols J.J. (2017). Concentrations of MUC16 and MUC5AC using three tear collection methods. Mol. Vis..

[7] Ellman G.L. (1959). Tissue sulfhydryl groups. Arch. Biochem. Biophys..

[8] Witko-Sarsat V., Friedlander M., Capeillère-Blandin C., Nguyen-Khoa T., Nguyen A.T., Zingraff J., Jungers P., Descamps-Latscha B. (1996). Advanced oxidation protein products as a novel marker of oxidative stress in uremia. Kidney Int..

[9] Erel O. (2004). A novel automated method to measure total antioxidant response against potent free radical reactions. Clin. Biochem..

[10] Erel O. (2005). A new automated colorimetric method for measuring total oxidant status. Clin. Biochem..

[11] Kosecik M., Erel O., Sevinc E., Selek S. (2005). Increased oxidative stress in children exposed to passive smoking. Int. J. Cardiol..

[12] Wong A.B., Wang M.T., Liu K., Prime Z.J., Danesh-Meyer H.V., Craig J.P. (2018). Exploring topical anti-glaucoma medication effects on the ocular surface in the context of the current understanding of dry eye. Ocul. Surf..

[13] Alm A. (2014). Latanoprost in the treatment of glaucoma. Clin. Ophthalmol..

[14] Jaenen N., Baudouin C., Pouliquen P., Manni G., Figueiredo A., Zeyen T. (2007). Ocular Symptoms and Signs with Preserved and Preservative-Free Glaucoma Medications. Eur. J. Ophthalmol..

[15] Chung S.H., Lee S.K., Cristol S.M., Lee E.S., Lee D.W., Seo K.Y., Kim E.K. (2006). Impact of short-term exposure of commercial eyedrops preserved with benzalkonium chloride on precorneal mucin. Mol. Vis..

[16] Kahook M., Noecker R. (2008). Quantitative analysis of conjunctival goblet cells after chronic application of topical drops. Adv. Ther..

[17] Baudouin C., Liang H., Hamard P., Riancho L., Creuzot-Garcher C., Warnet J.-M., Brignole-Baudouin F. (2008). The ocular surface of glaucoma patients treated over the long term expresses inflammatory markers related to both T-helper 1 and T-helper 2 pathways. Ophthalmology.

[18] Denoyer A., Godefroy D., Célérier I., Frugier J., Riancho L., Baudouin F., Rostène W., Baudouin C. (2012). A Comparison of the Effects of Benzalkonium Chloride on Ocular Surfaces between C57BL/6 and BALB/c Mice. Mucosal. Immunol..

[19] Potvin R., Makari S., Rapuano C. (2015). Tear film osmolarity and dry eye disease: A review of the literature. Clin. Ophthalmol..

[20] Labbé A., Terry O., Brasnu E., Went C.V., Baudouin C. (2012). Tear film osmolarity in patients treated for glaucoma or ocular hypertension. Cornea.

[21] Rath A., Eichhorn M., Träger K., Paulsen F., Hampel U. (2019). In vitro effects of benzalkonium chloride and prostaglandins on human meibomian gland epithelial cells. Ann. Anat..

[22] Seen S., Tong L. (2017). Dry eye disease and oxidative stress. Acta. Ophthalmol..

[23] Deng R., Hua X., Li J., Chi W., Zhang Z., Lu F., Zhang L., Pflugfelder S.C., Li D.-Q. (2015). Oxidative Stress Markers Induced by Hyperosmolarity in Primary Human Corneal Epithelial Cells. PLoS ONE.

[24] Bhatia R.P., Dash A., Dhawan S., Khanna H.D. (2010). Indirect evaluation of corneal apoptosis in contact lens wearers by estimation of nitric oxide and antioxidant enzymes in tears. Oman J. Ophthalmol..

[25] Ohashi Y., Dogru M., Tsubota K. (2006). Laboratory findings in tear fluid analysis. Clin. Chim. Acta.

[26] Openkova Y.Y., Korobeiynikova E.N., Rykin V.S., Vinkova G.A. (2013). The analysis of status of biochemical indicators in blood serum and lacrimal fluid in patients with primary open-angle glaucoma. Klin. Lab. Diagn..

[27] Baudouin C., Renard J., Nordmann J., Denis P., Lachkar Y., Sellem E., Rouland J., Jeanbat V., Bouée S. (2013). Prevalence and risk factors for ocular surface disease among patients treated over the long term for glaucoma or ocular hypertension. Eur. J. Ophthalmol..

[28] Junqueira V., Barros S., Chan S., Rodrigues L., Giavarotti L., Abud R., Deucher G. (2004). Aging and oxidative stress. Mol. Asp. Med..

[29] Jee D., Park M., Lee H.J., Kim M.S., Kim E.C. (2015). Comparison of treatment with preservative-free versus preserved sodium hyaluronate 0.1% and fluorometholone 0.1% eyedrops after cataract surgery in patients with preexisting dry-eye syndrome. J. Cataract. Refract. Surg..

[30] Oga T., Matsuoka T., Yao C., Nonomura K., Kitaoka S., Sakata D., Kita Y., Tanizawa K., Taguchi Y., Chin K. (2009). Prostaglandin F(2alpha) receptor signaling facilitates bleomycin-induced pulmonary fibrosis independently of transforming growth factor-beta. Nat. Med..

[31] Basu S. (2001). Raised levels of F2-isoprostanes and prostaglandin F2α in different rheumatic diseases. Ann. Rheum. Dis..

[32] Helmersson J., Larsson A., Vessby B., Basu S. (2005). Active smoking and a history of smoking are associated with enhanced prostaglandin F2α, interleukin-6 and F2-isoprostane formation in elderly men. Atherosclerosis.

[33] Helmersson J., Vessby B., Larsson A., Basu S. (2004). Association of Type 2 Diabetes With Cyclooxygenase-Mediated Inflammation and Oxidative Stress in an Elderly Population. Circulation.

[34] Basu S. (1999). Oxidative Injury Induced Cyclooxygenase Activation in Experimental Hepatotoxicity. Biochem. Biophys. Res. Commun..

[35] Basu S., Eriksson M. (1998). Oxidative injury and survival during endotoxemia. FEBS Lett..

[36] Claar D., Hartert T., Peebles R. (2014). The role of prostaglandins in allergic lung inflammation and asthma. Expert Rev. Respir. Med..

[37] Lopilly Park H.Y., Kim J.H., Lee K.M., Park C.K. (2012). Effect of prostaglandin analogues on tear proteomics and expression of cytokines and matrix metalloproteinases in the conjunctiva and cornea. Exp. Eye Res..

[38] Acera A., Rocha G., Vecino E., Lema I., Durán J.A. (2008). Inflammatory Markers in the Tears of Patients with Ocular Surface Disease. Ophthalmic Res..

[39] Messmer E.M., Lindenfels V.V., Garbe A., Kampik A. (2016). Matrix Metalloproteinase 9 Testing in Dry Eye Disease Using a Commercially Available Point-of-Care Immunoassay. Ophthalmology.

[40] Shetty R., Ghosh A., Lim R.R., Subramani M., Mihir K., Reshma A.R., Ranganath A., Nagaraj S., Nuijts R.M., Beuerman R. (2015). Elevated Expression of Matrix Metalloproteinase-9 and Inflammatory Cytokines in Keratoconus Patients Is Inhibited by Cyclosporine A. Invest. Ophthalmol. Vis. Sci..

[41] Reddy S., Sahay P., Padhy D., Sarangi S., Suar M., Modak R., Rao A. (2018). Tear biomarkers in latanoprost and bimatoprost treated eyes. PLoS ONE.

[42] Gomes J.A.P., Azar D.T., Baudouin C., Efron N., Hirayama M., Horwath-Winter J., Kim T., Mehta J.S., Messmer E.M., Pepose J.S. (2017). TFOS DEWS II iatrogenic report. Ocul. Surf..

[43] Cavet M.E., Harrington K.L., Vollmer T.R., Ward K.W., Zhang J.Z. (2011). Anti-inflammatory and anti-oxidative effects of the green tea polyphenol epigallocatechin gallate in human corneal epithelial cells. Mol. Vis..

[44] Li J., Deng R., Hua X., Zhang L., Lu F., Coursey T.G., Pflugfelder S.C., Li D.Q. (2016). Blueberry Component Pterostilbene Protects Corneal Epithelial Cells from Inflammation via Anti-oxidative Pathway. Sci. Rep..

